# Targeted temperature control following traumatic brain injury: ESICM/NACCS best practice consensus recommendations

**DOI:** 10.1186/s13054-024-04951-x

**Published:** 2024-05-20

**Authors:** Andrea Lavinio, Jonathan P. Coles, Chiara Robba, Marcel Aries, Pierre Bouzat, Dara Chean, Shirin Frisvold, Laura Galarza, Raimund Helbok, Jeroen Hermanides, Mathieu van der Jagt, David K. Menon, Geert Meyfroidt, Jean-Francois Payen, Daniele Poole, Frank Rasulo, Jonathan Rhodes, Emily Sidlow, Luzius A. Steiner, Fabio Silvio Taccone, Riikka Takala

**Affiliations:** 1 Department of Medicine, BOX 1 Addenbrooke’s Hospital, University of Cambridge, Long Road, Cambridge, CB2 0QQ UK; 2 Department of Anaesthesia and Critical Care, Cambridge University Hospitals NHS Foundation Trust, Cambridge, UK; 3 IRCCS Policlinico San Martino, Genoa, Italy; 4 Department of Intensive Care, Maastricht University Medical Center+, Maastricht, The Netherlands; 5 School of Mental Health and Neurosciences, University Maastricht, Maastricht, The Netherlands; 6 Inserm U1216, Department of Anesthesia and Critical Care, CHU Grenoble Alpes, Grenoble Institute Neurosciences, Université Grenoble Alpes, 38000 Grenoble, France; 7 Medical Intensive Care Unit, Saint-Louis Teaching Hospital, Paris, France; 8 Department of Anaesthesia and Intensive Care, University Hospital of North Norway, Tromsö, Norway; 9 Department of Clinical Medicine, UiT the Arctic University of Norway, Tromsö, Norway; 10 Department of Intensive Care, Hospital General Universitario de Castellón, Castellón de la Plana, Spain; 11 Department of Neurology, Kepler University Hospital, Johannes Kepler University, Linz, Austria; 12 Clinical Research Institute for Neuroscience, Johannes Kepler University, Linz, Austria; 13 Department of Anaesthesiology, Amsterdam UMC, Amsterdam, The Netherlands; 14 Department of Intensive Care Adults, Erasmus MC, University Medical Centre, Rotterdam, The Netherlands; 15 Department and Laboratory of Intensive Care Medicine, University Hospitals Leuven, Leuven, Belgium; 16 Anesthesia and Intensive Care Operative Unit, S. Martino Hospital, Belluno, Italy; 17 Spedali Civili University Hospital of Brescia, Brescia, Italy; 18 Department of Anaesthesia, Critical Care and Pain Medicine, Royal Infirmary of Edinburgh, University of Edinburgh, Edinburgh, UK; 19 Page and Page Healthcare Communications, London, UK; 20 University Hospital Basel, Department of Clinical Research, University of Basel, Basel, Switzerland; 21 Department of Intensive Care, Brussels University Hospital, Brussels, Belgium; 22 Université Libre de Bruxelles, Brussels, Belgium; 23 Perioperative Services, Intensive Care Medicine and Pain Management, Turku University Hospital, Turku, Finland; 24 Department of Anaesthesiology and Intensive Care, University of Turku, Turku, Finland

**Keywords:** Traumatic brain injury, Targeted temperature control, Fever, Intracranial pressure, Normothermia

## Abstract

**Aims and scope:**

The aim of this panel was to develop consensus recommendations on targeted temperature control (TTC) in patients with severe traumatic brain injury (TBI) and in patients with moderate TBI who deteriorate and require admission to the intensive care unit for intracranial pressure (ICP) management.

**Methods:**

A group of 18 international neuro-intensive care experts in the acute management of TBI participated in a modified Delphi process. An online anonymised survey based on a systematic literature review was completed ahead of the meeting, before the group convened to explore the level of consensus on TTC following TBI. Outputs from the meeting were combined into a further anonymous online survey round to finalise recommendations. Thresholds of ≥ 16 out of 18 panel members in agreement (≥ 88%) for strong consensus and ≥ 14 out of 18 (≥ 78%) for moderate consensus were prospectively set for all statements.

**Results:**

Strong consensus was reached on TTC being essential for high-quality TBI care. It was recommended that temperature should be monitored continuously, and that fever should be promptly identified and managed in patients perceived to be at risk of secondary brain injury. Controlled normothermia (36.0–37.5 °C) was strongly recommended as a therapeutic option to be considered in tier 1 and 2 of the Seattle International Severe Traumatic Brain Injury Consensus Conference ICP management protocol. Temperature control targets should be individualised based on the perceived risk of secondary brain injury and fever aetiology.

**Conclusions:**

Based on a modified Delphi expert consensus process, this report aims to inform on best practices for TTC delivery for patients following TBI, and to highlight areas of need for further research to improve clinical guidelines in this setting.

**Supplementary Information:**

The online version contains supplementary material available at 10.1186/s13054-024-04951-x.

## Introduction

Traumatic brain injury (TBI) is a complex and heterogeneous disease, and a major cause of death and disability globally [1–3]. Amongst other common neurological diseases, TBI is estimated to have the highest prevalence and incidence, impacting up to 60 million people worldwide annually and representing a substantial public health burden [4].

TBI is defined as an alteration in brain function or other evidence of brain pathology caused by an external force [5], and requires immediate and sustained management strategies to optimise clinical outcome. The injury processes that follow from a TBI are divided into two stages: primary and secondary [6], where primary injury refers to the damage caused by the original physical impact, which can trigger a pathophysiological cascade resulting in secondary injury with deleterious effects on neurological outcome and survival [7, 8]. In order to prevent or mitigate secondary injury, immediate treatment following severe TBI focuses on the prevention of further brain damage. As the brain remains susceptible to secondary injury from processes that extend beyond the zone of primary injury such as ischaemia, oedema, herniation, seizures and altered metabolism [9], immediate treatment following severe TBI focuses on prevention or mitigation of such injury. This is achieved through the control of intracranial pressure (ICP), and prompt treatment of systemic insults such as hypoxia, hypercapnia, and systemic hypotension [10].

In the neuro-intensive care unit (NICU), fever is a prevalent occurrence with heterogenous underlying causes, and it may contribute to secondary injury. Across patients with TBI, subarachnoid haemorrhage and stroke [11–13], hyperthermia has been found to increase the risk of complications and is believed to be associated with unfavourable clinical outcome including death [9, 11, 14, 15].

Targeted temperature control (TTC) is a complex intervention that aims to control body or brain temperature to prevent further brain injury and improve neurological outcome [9]. The term TTC may refer to different degrees of temperature control, from fever prevention, maintenance of normothermia to the induction of hypothermia, at different levels [9, 16]. In TBI, TTC can be used to modulate a range of important physiological parameters such as cerebral metabolism and ICP. However, its role in improving long-term outcome, as well as the appropriate indications, targets and duration of TTC in severe or moderate TBI are currently unknown.

This work aims to utilise a Delphi approach to develop best-practice consensus recommendations from international experts for the real-world application of TTC in severe TBI with ICP guided treatments.

## Methods

### Review of the literature and evidence quality assessment

Statements and questions were informed by a systematic review of the literature, which identified observational studies, meta-analyses and randomised controlled trials (RCTs) relevant to the topics under discussion. This review search focused on evidence released since 2013. Following this first review, the methodology group of ESICM conducted an independent systematic review of the literature, considering only published RCTs regarding TTC in TBI patients with ICP monitoring. This review confirmed the paucity of RCTs and the substantial clinical heterogeneity between them, which precluded meta-analytical combination. The outputs from the reviews were shared with the expert panel members ahead of the Delphi process. A detailed reporting of the literature reviews is provided as Additional files 1 and 4.

### Participants

The 18 expert attendees for the Delphi process were chosen from members of three professional societies: the Neuro Anaesthesia and Critical Care Society (NACCS), the European Society of Intensive Care Medicine (ESICM), and the European Society of Anaesthesiology and Intensive Care (ESAIC). Selection was based on a documented history of publications in the fields of traumatic brain injury and/or targeted temperature management, as well as their established professional profiles and expertise as leading intensive care practitioners in teaching university hospitals. We endeavoured to ensure balanced representation, covering the geographic areas of the EU, Switzerland, and the UK.

### Delphi rounds

**Fig. 1:**
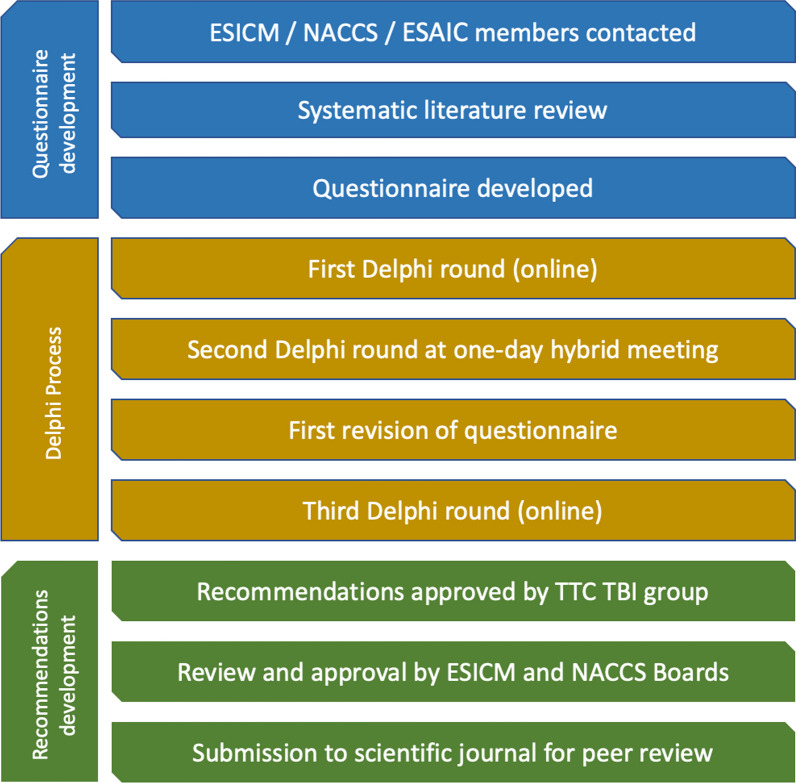
Summary of the Delphi process. *ESAIC* European Society of Anaesthesiology and Intensive Care, *ESICM* European Society of Intensive Care Medicine, *NACCS* Neuro Anaesthesia and Critical Care Society

A modified Delphi consensus method was employed, involving a combination of an online survey (Round 1), a face-to-face meeting (Round 2), an additional online survey containing the refined questions from the previous steps, (Round 3) and post-meeting reviews of the consensus results. The questions asked at Round 1 can be found in the Additional file 2, and the results following Round 3 are shown in Table 1. Round 1 was conducted via the SmartSurvey® online platform, and Round 2 was held as a hybrid meeting in London, UK, on Tuesday 10th October 2023. AL acted as Chair, with an independent facilitator (ES) moderating the meeting. After the results from the final survey of Round 3 were received, the recommendations and final manuscript were developed, with documents shared by e-mail and feedback collected independently from each participant by the facilitator. The predefined agreed cut-off for strong consensus was to have ≥ 16 out of 18 (≥ 88%) of panel members in agreement, and for moderate consensus was to have ≥ 14 out of 18 (≥ 78%) of panel members in agreement. The Delphi methodology and process was adopted from the manuscript published by Lavinio et al. [17]. In a Delphi process, conflicting opinions are addressed through a structured framework that promotes consensus-building among experts. Initially, participants are asked to provide their views anonymously, which are then summarised and shared with the group. This approach facilitates open and unbiased input, as the anonymity helps mitigate the influence of dominant personalities or hierarchical pressures. When conflicting opinions emerge, they are documented and presented back to the participants, along with any common ground that has been identified. In subsequent rounds, individuals are encouraged to reconsider their positions in light of the collective feedback, which often leads to a convergence of opinions. If discrepancies persist, these are explored through further iterative rounds, with an emphasis on clarifying rationale and seeking areas of agreement. The Delphi method's iterative nature, combined with the feedback mechanism, effectively manages conflicting opinions by fostering a gradual move towards consensus, or at least a clearer understanding of the points of divergence. The process for the Delphi panel and subsequent manuscript development is visualised in Fig. 1. A detailed overview of the iterative Delphi process is provided in the Additional files 2 and 3.

**Table 1:** Summary of panel recommendations

Recommendation	Level of consensus	Stage reached
*Pathophysiology*		
Temperature measurement and control is an essential aspect of high-quality care in patients with severe traumatic brain injury (TBI)	Strong consensus (100%)	Round 3
In patients with impending cerebral herniation, temperature control is essential	Strong consensus (89%)	Round 3
*Monitoring*		
Continuous temperature monitoring is preferable over intermittent temperature measurements in patients with severe TBI	Strong consensus (100%)	Round 1
Monitoring core temperature (e.g., bladder, oesophageal, brain) is strongly recommended over measuring or monitoring superficial temperature (e.g., skin, tympanic) in severe TBI	Strong consensus (94%)	Round 3
Monitoring brain temperature is recommended in addition to monitoring core systemic temperature as a therapeutic target	*No consensus (61%)*	
When brain temperature monitoring is not immediately available, alternative sources of core temperature (oesophageal, bladder, intravascular) are acceptable	Strong consensus (89%)	Round 3
When brain temperature monitoring is in place, it is advisable to also assess core temperature	Strong consensus (100%)	Round 3
*ICP*		
Temperature control is a key component of intracranial pressure (ICP) management in severe TBI cases	Strong consensus (100%)	Round 1
Controlled normothermia (i.e., target core temperature 36.0–37.5 °C) should be included as an addition to the Tier 1 and Tier 2 treatments defined within the SIBICC 2019 guidelines	Moderate consensus (83%)	Round 3
Therapeutic hypothermia (i.e., target core temperature ≤ 36.0 °C) should be considered in cases where tier 1 and 2 treatments (as per SIBICC guidance) have failed to control ICP	Moderate consensus (83%)	Round 3
If hypothermia is considered to control ICP, target temperature should be managed as close to physiological temperature as possible	Strong consensus (94%)	Round 3
In patients with impending brain herniation, therapeutic hypothermia should be considered as a temporising strategy, and should be induced rapidly	*No consensus (61%)*	
In patients with impending herniation awaiting surgical evacuation or decompression, the lowest target core temperature at which hypothermia should be initiated as a short-term temporising strategy is	*No consensus*^a^ (61%—35.0 °C; 17%—34.0 °C; 6%—33.0 °C; 17%—N/A)	Round 3
In patients with exhausted intracranial volume buffering reserve and labile ICP with occasional spikes > 25 mmHg, the lowest target core temperature that a medium term ICP-control strategy should be implemented at is	*No consensus*^a^ (56%—35.0 °C; 33%—34.0 °C; 6%—33.0 °C; 6%—N/A)	Round 3
In tier 3 treatment in SIBICC guidelines, before considering decompressive craniectomy, hypothermia (< 36.0 °C) should be attempted	*No consensus (44%)*	
Before considering barbiturate burst suppression, hypothermia (< 36.0 °C) should be attempted	*No consensus (61%)*	
*Fever*		
Uncontrolled fever (neurogenic or secondary to inflammation or infection) can precipitate secondary brain injury in patients with severe TBI	Strong consensus (100%)	Round 3
Fever control is recommended in patients with severe TBI who have seizures or are perceived to be at high risk of seizures	Strong consensus (94%)	Round 3
Fever increases the risk of intracranial hypertension in patients with severe TBI	Strong consensus (94%)	Round 3
Neurogenic fever (core temperature > 37.5 °C driven by neurological dysregulation in the absence of sepsis or clinically significant inflammatory process) is relatively common in traumatic brain injury cases, and it should be promptly detected and treated (i.e., with controlled normothermia targeting 36.0–37.5 °C), irrespective of ICP	Moderate consensus (83%)	Round 3
Controlled normothermia should be considered when pyrexia is secondary to sepsis or inflammatory processes, and when the patient is perceived to be at risk of secondary brain injury, especially in the acute phase of TBI	Strong consensus (94%)	Round 3
In patients with severe TBI who are sedated and ventilated, controlled normothermia, irrespective of ICP, should be initiated reactively when fever is detected	Strong consensus (94%)	Round 3
When neurogenic fever is detected in TBI cases, controlled normothermia should be continued for as long as the brain remains at risk of secondary brain damage	Strong consensus (89%)	Round 3
*Hypothermic TTC induction*		
It is recommended that the rapid induction of hypothermia in TBI cases should be achieved with automated feedback-controlled temperature management devices	Strong consensus (89%)	Round 3
It is advisable that neurotrauma ICUs should stock readily available NaCl solutions of different concentrations stored at ice-cold temperature for the management of intracranial hypertension crises	No consensus *(50%)*	
*TTC maintenance*		
An automated feedback-controlled TTC device that enables precise temperature control is desirable for the initiation of TTC and maintenance at target temperature in patients with severe TBI	Strong consensus (100%)	Round 1
The maximum temperature variation that a patient should experience during normothermia is less than or equal to ± 0.5 °C per hour and ≤ 1 °C per 24-h period	Moderate consensus (78%)	Round 3
When hypothermia is indicated, treatment should be continued for as long as the brain is considered to be at risk of secondary brain injury	Strong consensus (89%)	Round 3
*Rewarming following hypothermic TTC*		
Obtaining an interval scan and/or an alternative assessment of intracranial compliance, in addition to the absolute number of ICP, is recommended before rewarming	Strong consensus (89%)	Round 3
When rewarming a patient from therapeutic hypothermia, rewarming should be controlled by an automated feedback-controlled TTC device and should not exceed 1.0 °C per 24-h period	*No consensus (44%)*	
Rebound hyperthermia should be prevented whenever possible or promptly treated in cases when the brain is perceived to be at risk of secondary brain injury	Strong consensus (100%)	Round 3
*Shivering*		
It is important to assess, document and manage shivering in severe TBI patients	Strong consensus (100%)	Round 3
Whenever ICP is labile and shivering is detected, neuromuscular blockers should be considered after ensuring appropriate depth of sedation	Strong consensus (94%)	Round 3
In self-ventilating patients in the subacute phase of severe TBI, an individualised risk–benefit assessment should be undertaken regarding the indications of controlled normothermia	Strong consensus (100%)	Round 3
Permissive hyperthermia should be considered in cases where risk of secondary brain injury resulting from pyrexia is thought to be low, and when shivering cannot be controlled with first line treatments such as NSAIDs, opiates, magnesium or counter warming	Moderate consensus (83%)	Round 3
*Auditing*		
Time within target range, burden of fever and similar metrics can be considered as indicators of quality of temperature management	Strong consensus (94%)	Round 3

^a^Questions 16 and 17 explored what the lowest target temperature should be when therapeutic hypothermia is considered as a short-term and as a medium term ICP-control measure. Whilst no consensus was achieved, the majority of experts indicated 35.0 °C as the lowest temperature in both scenarios. The breakdown of responses to questions 16 and 17 is provided in the table

### Definitions

To guide discussions during the Delphi process, clinical terms were defined with the values as shown below.

**Table:** 

Clinical term	Definition
Mild hypothermia	Core temperature 34.0–36.0 °C
Therapeutic hypothermia	Core temperature < 36.0 °C
Controlled normothermia	Core temperature 36.0–37.5 °C
Fever	Core temperature > 37.5 °C

### Declarations and conflicts of interest

The face-to face meeting in London was supported by Becton, Dickinson and Company (“BD”) through the provision of travel costs, meeting space and refreshments. Representatives from BD were allowed to silently observe the conference, without any interaction with the panellists or the process. No donors or other outside parties influenced any portion of these recommendations. There was no industry input into recommendation development, and no panel member received honoraria for their involvement. Panellists completed conflict of interest forms relevant to TBI management. There were no conflicts mandating recusal of any participant. No funding was provided by the societies involved.

## Results

The results of the final consensus are presented in Table 1. We highlight and expand upon statements in which consensus was reached in the discussion section. Some consideration is added to statements in which consensus was not reached, proposing them as potential areas for valuable future research.

## Discussion

To date, there is a lack of definitive evidence regarding the use of TTC with an automated feedback-controlled device for managing temperature in severe TBI. This underlines the importance of consensus discussion in identifying areas of uncertainty where evidence is lacking, and in encouraging harmonised care delivery across different settings.

### Pathophysiology

(i) Temperature measurement and control is an essential aspect of high-quality care in patients with severe TBI
(ii) In patients with impending cerebral herniation, temperature control is essential

As an introduction to the discussions, the group debated the recommendation for temperature measurement and control following severe TBI and, after extensive discussion, concluded that core temperature measurement and control is essential for the provision of high-quality care, especially in patients perceived to be at high risk of secondary brain injury. Noting the phrasing of ‘temperature control’ in the recent guidelines for temperature control following cardiac arrest [18], the group agreed that as an entry point into high-quality care following TBI, the notion of temperature measurement and control is key, opening the door to the full practice of targeted temperature management. This nuanced phrasing was intended to set the scene for the group’s work, with the specifics of the TTC process such as temperature ranges and duration of control being addressed throughout the remainder of the discussions.

Highlighting the wealth of physiological data available on the management of temperature in stroke and cardiac arrest, the group noted that the guidelines for temperature management in TBI are less specific. Fundamentally, the group agreed that high-quality TBI care does include monitoring temperature and implementing some form of temperature control, recognising its potential role in optimising outcome. The group highlighted the importance of treatment titration based on an individualised risk–benefit assessment and stratification. In particular, it was noted that in patients with exhausted intracranial compensatory reserve and at risk of cerebral herniation or ischaemia—there exists an extreme susceptibility to secondary brain injury precipitated by suboptimal temperature control.

Cerebral herniation is a life-threatening event that requires early diagnosis and prompt management in order to prevent irreversible pathological cascades that can lead to death [19]. Increases in brain temperature have been linked to a linear rise in ICP, with the relationships between temperature, ICP and cerebral perfusion pressure (CPP) becoming more apparent with rapid temperature changes. The impact of temperature on ICP supports the recommendation from the group that temperature control is an essential aspect of care in patients at risk of herniation [20]. The group agreed that while control of ICP and prevention of herniation were important reasons for TTC in TBI, benefits of TTC in the acute phase of TBI also extended to patients without intracranial hypertension.

During the discussions the group highlighted that different pathologies often dictate different patient management. For example, patients in whom fluctuations in ICP are well-tolerated (e.g., patients with high intracranial compliance) will be managed differently to patients with obliterated basal cisterns, obliterated cortical sulci, and midline shift (e.g., intracranial mass effect). In patients with exhausted intracranial volume-buffering reserve, strict control of physiological parameters such as CO_2_ and temperature, is strongly recommended.

### Monitoring

1. Continuous temperature monitoring is preferable over intermittent temperature measurements in patients with severe TBI.
2. Monitoring core temperature (e.g., bladder, oesophageal, brain) is strongly recommended over measuring or monitoring superficial temperature (e.g., skin, tympanic) in severe TBI.
3. When brain temperature monitoring is in place, it is advisable to assess an additional source of core temperature monitoring (i.e. oesophageal, bladder).

The group widely agreed, in line with supporting literature, that continuous temperature monitoring is preferable over intermittent temperature measurements with severe TBI. Intermittent monitoring and recording of temperature can result in large fluctuations in temperature being missed, as highlighted by supporting literature investigating the use of TTC following cardiac arrest, TBI and stroke [17, 21, 22].

Discussions amongst the group drew attention to the fact that inaccurately measured temperatures can negatively impact patient care and outcome. Several temperature monitoring sites are available for TTC, and the group widely agreed that core temperature measurements, i.e., bladder and oesophageal sites, are strongly preferred over superficial measurements such as those taken at skin and tympanic sites. Following acknowledgement of their limitations [23], bladder and oesophageal were singled out as favoured core temperature measurements. The group acknowledged the widespread use of oesophageal probes due to their relative ease of insertion and the challenges of finding MRI compatible bladder probes. Confirmation of preference between the two was acknowledged as being beyond the scope of the group due to these nuances. Rectal temperature monitoring was widely regarded as impractical for reasons such as the lag time and a high rate of dislocation [16, 23]. Peripheral sites were unanimously deemed to be insufficiently accurate to guide temperature treatment [16].

Some panel members argued that monitoring target organ (i.e. brain) temperature could add a layer of clinical safety, improve pathophysiological understanding and allow selective and individualised titration of treatment (i.e. selective brain cooling). It was, however, agreed by the group that more research is needed into optimum methods for measuring brain temperature and its interpretation from both a clinical and resource-availability perspective. In particular, it was highlighted that temperature thresholds for harm are less well defined for brain temperature than core temperature. When brain temperature monitoring is available and in place, the group advised that core temperature should also be assessed with bladder or oesophageal probes since this is part of routine practice and has been studied to a greater extent than brain temperature. The group noted the importance of having a dual source of temperature monitoring when using automated TTC devices to reduce the risk of probe malfunction and subsequent over or undercooling [24].

After TBI, brain temperature has often been shown to be higher than systemic temperature and can vary independently, with literature noting a difference of as much as 2 °C depending on the individual characteristics of brain pathology and/or probe location, making a consistent and accurate link between the two challenging and possibly inaccurate [25, 26]. The group highlighted that targeting brain temperature may allow precise titration of treatment dose, including titration of selective brain cooling with brain temperature management technologies, theoretically reducing side effects associated with systemic hypothermia, whilst delivering neuroprotection and brain temperature management. However, it was concluded that further research is needed in this regard and that not enough evidence exists to support practical recommendations.

### ICP management

1. Temperature control is a key component of ICP management in severe TBI.
2. Controlled normothermia (i.e., target core temperature 36.0–37.5 °C) should be included as an addition to the Tier 1 and Tier 2 treatments defined within the Seattle International Severe Traumatic Brain Injury Consensus Conference (SIBICC) 2019 guidelines.
3. Therapeutic hypothermia (i.e., target core temperature ≤ 36.0 °C) should be considered in cases where tier 1 and 2 treatments (as per SIBICC guidance) have failed to control ICP.
4. If hypothermia is considered to control ICP, target temperature should be managed as close to normothermia as possible.

ICP monitoring remains a critical component in the management of severe TBI [27, 28]. The group unanimously agreed that temperature control is a key aspect of managing ICP, highlighting that an increase in temperature can lead to an increase in cerebral metabolism and augmented cerebral blood flow, and a simultaneous increase in cerebral blood volume. In cases of exhausted compensatory mechanisms, these factors can precipitate intracranial hypertension [20], which in turn can have a deleterious effect on overall outcome.

Because there is often no single pathophysiological pathway of ICP elevation, its management is complex. The most recent versions of the Brain Trauma Foundation TBI guidelines do not contain treatment protocols, in part due to a lack of solid evidence around the relative efficacy of available interventions [27]. To address this, the Seattle International Severe Traumatic Brain Injury Consensus Conference (SIBICC) developed a consensus-based practical algorithm for tiered management of severe TBI guided by ICP measurements [28].

**Fig. 2:**
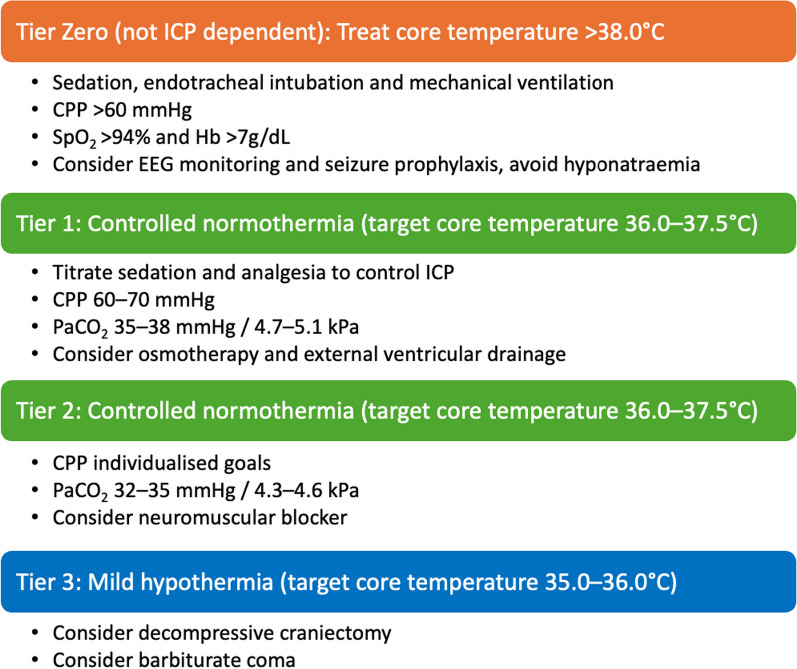
Intracranial pressure management algorithm for severe TBI edited from SIBICC 2019 [28]. * Including TTC in tiers 1 and 2 is the suggested addition from the TTC-TBI group to the original SIBICC tiers (green bars). *When possible, the lowest tier should be used. It is not necessary to use all modalities in a previous tier before moving to the next tier. Consider repeat CT and surgical options for space occupying lesions. *CPP* cerebral perfusion pressure, *CT* computed tomography, *EEG* electroencephalography, *Hb* haemoglobin, *kPa* kilopascal, *mmHg* milimetre of mercury, *PaCO*_*2*_ arterial partial pressure of carbon dioxide, *SpO*_*2*_ arterial oxygen saturation

One of the most impactful outcomes from this consensus meeting was the acknowledgement of the essential role of temperature control for ICP management in severe TBI, and the recommendation that controlled normothermia (i.e., target core temperature 36.0–37.5 °C) should be considered in addition to Tier 1 and Tier 2 treatments. The group was keen to harmonise this output with SIBICC by suggesting a more aggressive and specific management with the addition of controlled normothermia in Tiers 1 and 2, adding a layer of clinical safety beyond merely the avoidance of fever over 38.0 °C in Tier 0, as shown in Fig. 2. In cases when hypothermia is considered (i.e., SIBICC Tier 3), the group recommended that target temperature be managed as close to normothermia as possible, based on an individualised risk–benefit assessment [29].

No consensus was reached on whether hypothermia was a viable temporising strategy in patients with impending cerebral herniation, in patients awaiting haematoma evacuation or decompression, or before consideration of barbiturate coma. Whilst the group acknowledged that therapeutic hypothermia can be effective in reducing ICP, there was no consensus on whether this could be induced rapidly enough in these circumstances, and it was felt that insufficient evidence was available to provide pragmatic recommendations on its indication in these extreme clinical circumstances.

Whilst the majority of experts indicated 35.0 °C as the lowest target temperature to be considered in these circumstances, no consensus was reached. The discussion highlighted that insufficient evidence exists to support practical recommendations and highlighted the importance of an individualised risk–benefit assessment. It was also noted that centres might have a varying degree of familiarity with different therapeutic options, including ease of access to neurosurgical options (i.e. ventricular drainage, decompression) and this may have an impact on clinician preference for hypothermia as a temporising therapeutic modality.

The group also discussed the indication of barbiturates in the context of ICP control following severe TBI, not reaching consensus on whether therapeutic hypothermia should be attempted before considering barbiturates. The group noted that both barbiturate-induced burst-suppression and therapeutic hypothermia have distinctive side effects and concluded that no recommendations for standard clinical practice could be made beyond what was already stated in SIBICC guidance.

### Fever

1. Neurogenic fever (core temperature > 37.5 °C) driven by neurological dysregulation in the absence of sepsis or a clinically significant systemic inflammatory process is relatively common in TBI, and it should be promptly detected and treated (i.e., with controlled normothermia targeting 36.0 °C to 37.5 °C), irrespective of ICP level.
2. Controlled normothermia should be considered when pyrexia is secondary to sepsis or inflammatory processes, and when the patient is perceived to be at risk of secondary brain injury, especially in the acute phase of TBI.
3. Uncontrolled fever (neurogenic or secondary to inflammation or infection) can precipitate secondary brain injury in patients with severe TBI.

It was widely agreed that neurogenic fever, defined here as core temperature > 37.5 °C driven by neurological dysregulation in the absence of sepsis or a clinically significant inflammatory process is common in intensive care and it has been found to be associated with an increased risk of complications and unfavourable outcome [9, 14, 15]. In the setting of neurogenic fever developing in comatose patients with acute traumatic encephalopathies, controlled normothermia targeting 36.0–37.5 °C was recommended in tier 1 and 2 of the ICP management algorithm.

Correctly differentiating central fever against fever of infectious origin is both challenging and clinically important due to the impact of failing to identify a treatable condition, the negative consequences of antibiotic overuse, and the detrimental effect of hyperthermia on brain-injured patients [17, 30, 31]. However, the group noted that physiological processes such as brain metabolic rate of oxygen, CO_2_ control, brain tissue oxygenation (P_bt_O_2_) and ICP are directly related to temperature, and that the deleterious effects and likelihood of secondary injury may occur irrespective of whether temperature is raised due to infection or impaired thermoregulation. This therefore highlights the need for acute management of temperature regardless of the source of the pyrexia, although added focus must be placed on the management of nuanced patient characteristics such as those with severe TBI with impending herniation and/or obliterated basal cisterns, as opposed those with low ICP and preserved intracranial compliance.

In line with current research [9, 11, 32], it was agreed that the development of fever is common in TBI cases, and that it can precipitate secondary brain injury and adversely affect patient outcome. It is therefore of utmost importance to prevent or promptly treat fever when detected. The group agreed that while some degree of controlled pyrexia may be allowed during the subacute phase of disease, ‘uncontrolled’ fever requires urgent management in the acute phase as long as the patient is still perceived to be at significant risk of secondary brain injury.

1. Fever control is recommended in patients with severe TBI who have seizures or are perceived to be at high risk of seizures.
2. In patients with severe TBI who are sedated and ventilated, controlled normothermia, irrespective of ICP, should be initiated reactively when fever is detected.
3. When neurogenic fever is detected in TBI cases, controlled normothermia should be continued for as long as the brain remains at risk of secondary brain damage.

The group strongly recommended that fever control and controlled normothermia are of particular relevance in patients perceived to be at high risk of seizures and, more in general, secondary brain injury. The assessment of whether an individual patient should be considered ‘at risk of seizures’ or ‘at risk of secondary brain injury’ remains the responsibility of the managing physician. The group defined risk factors for seizures as a history of seizures, the presence of temporal contusions or depressed skull fractures. Features associated with a higher ‘risk of secondary brain injury’ included labile ICP, obliterated basal cisterns, midline shift or subfalcine herniation, and other signs of exhausted intracranial volume buffering reserve. While no consensus was reached on a specific temperature range to target during controlled normothermia, the group agreed that the reactive initiation of temperature control was important in sedated and ventilated TBI patients, with agreement on a pragmatic setting of a target core temperature range of 36.0–37.5 °C to accommodate expected fluctuations of ± 0.5 °C while avoiding spikes over 38.0 °C [28].

### Hypothermic TTC induction

1. It is recommended that the rapid induction of hypothermia in traumatic brain injury cases should be achieved with automated feedback-controlled temperature management devices.

In line with current research [17], the group widely agreed on the reactive use of an automated feedback-controlled device for the application of optimal TTC. The TTC process can be divided into three phases: induction, maintenance, and rewarming [9, 16]. As explained in existing literature, varying availability of devices and financial aspects may dictate choice, and while non-automated methods of temperature control are cheaper and easier to apply, the level of control offered is poor and their use should be limited to the induction phase, as adjuncts to automated devices. [17, 33] Whilst antipyretics such as acetaminophen (paracetamol) or nonsteroidal anti-inflammatory drugs (NSAIDs) are widely acknowledged in intensive care unit (ICU) settings for their role in fever management, it is recognised that in the context of severe TBI, the efficacy of antipyretics in controlling fever and minimising temperature variability is limited. The application of therapeutic hypothermia requires constant monitoring of core body temperature in order to achieve an accurate target temperature during induction to prevent overcooling, to assess variations during the maintenance phase, and to ensure a steady, controlled rewarming phase [16].

There was no agreed recommendation from the group as to whether ICUs should stock readily available ice-cold NaCl solutions of different concentrations for the management of ICP crises, citing a lack of clear evidence to draw upon. The group did however highlight the fact that the rapid infusion of ice-cold saline is an inexpensive and readily available option for lowering core body temperature [9], with the rapidity of response to ice-cold infusions being regarded as a valuable aspect of TTC induction.

### TTC maintenance

1. An automated feedback-controlled TTC device that enables precise temperature control is desirable for the initiation of TTC and maintenance at target temperature in patients with severe TBI.
2. The maximum temperature variation that a patient should experience during normothermia is less than or equal to +/− 0.5 °C per hour and ≤ 1 °C per 24-hperiod

(3) When hypothermia is indicated, treatment should be continued for as long as the brain is considered to be at risk of secondary brain injury.

Automated feedback-controlled devices for TTC are powerful tools, encouraging the delivery of quality care and aiming to improve neurological outcome [13, 17], minimising the chances of temperature variability. Temperature variability is the deviation of patient temperature outside of the goal, typically reported as mean deviation or percent of time outside of target [9]. The group noted that there is a level of pragmatism to be adopted in TTC maintenance, discussing that while more time spent in fever can negatively impact neurological outcome, fluctuations in temperature may also affect outcome [17], and consensus was reached on the importance of maintaining temperature at as consistent a level as possible with the group settling on a fluctuation range of less than or equal to ± 0.5 °C per hour and ≤ 1 °C per 24-h period. In instances where an automated feedback-controlled device is not available, the group noted the importance of increased staff awareness of patient status to ensure fluctuations outside of this range are appropriately managed. The group highlighted that a dedicated protocol for sedation, analgesia and shivering management might be helpful to ensure consistent application of optimal TTC.

The group agreed that when indicated, hypothermia should be continued for as long as the individual practitioner considers the brain to be at risk of secondary injury. These considerations were supported with a suggestion that it should be maintained for as short a time as possible.

### Rewarming following hypothermic TTC

1. Obtaining an interval scan and/or an alternative assessment of intracranial compliance, in addition to the absolute number of ICP, is recommended before rewarming.
2. Rebound hyperthermia should be prevented whenever possible or promptly treated in cases when the brain is perceived to be at risk of secondary brain injury.

In cases in which the patient is being rewarmed from therapeutic hypothermia (core temperature lower than 36.0 °C), the group agreed that once ICP has been maintained within controlled limits and de-escalation of treatment intensity is considered, it is sensible to ensure the patient has sufficient intracranial volume buffering reserve through the use of an interval scan and/or an alternative measure of intracranial compliance, before commencing the rewarming process. The group also noted the high prevalence and potential risks associated with rebound hyperthermia when TTC is discontinued following therapeutic hypothermia, highlighting the importance of continued vigilance and careful temperature control in the rewarming phase.

Whilst no consensus was reached on recommended rewarming rates, the group agreed that controlled rewarming with an automated feedback-controlled device may reduce the risk of rapid temperature variations and rebound pyrexia that can precipitate secondary brain injury and compromise care [16, 33]. The group highlighted how controlled rewarming may improve the ability of clinicians to more effectively control important inter-dependent clinical variables such as PaCO_2_, ventilation settings and depth of sedation.

### TTC for shivering

1. It is important to assess, document and manage shivering in severe TBI patients.
2. Whenever ICP is labile and shivering is detected, neuromuscular blockers should be considered after ensuring appropriate depth of sedation.
3. In self-ventilating patients in the subacute phase of severe TBI, an individualised risk–benefit assessment should be undertaken regarding the strict indications of controlled normothermia.
4. Permissive hyperthermia should be considered in cases where risk of secondary brain injury resulting from pyrexia is thought to be low, and when shivering cannot be controlled with first line treatments such as NSAIDs, opiates, magnesium or counter warming.

In line with current literature, it was widely agreed that shivering should be managed in patients following severe TBI. Shivering can reduce brain tissue oxygenation leading to cerebral metabolic stress, which may therefore negate the neuroprotective benefits of TTC [9, 34–36].

Titration of sedation and the use of neuromuscular blocking agents provides intensivists with readily available and effective options for shivering control in critically ill patients [37]. To ensure appropriate and effective use however, treating staff must be aware of the nuances of selecting the correct agent, monitoring the depth of neuromuscular blockade, and ensuring adequate skeletal muscle recovery once therapy with neuromuscular blockers has ceased. In cases of shivering when ICP is labile, the group agreed in line with current literature that ensuring depth of sedation before administering neuromuscular blockers is of utmost importance [37, 38]. When using pharmacologic agents for shivering management, treating staff must consider potential pharmacokinetic and pharmacodynamic variation and monitor for efficacy (i.e. shivering control) and safety (i.e. adverse events and drug-drug interactions) [9].

The group agreed that in patients who are perceived to be at relatively lower risk of secondary brain injury (i.e. self-ventilating patients in the sub-acute phase of severe TBI), permissive hyperthermia may be considered over TTC, especially if the latter therapeutic option would require sedation or other invasive interventions. The group agreed that an individualised risk–benefit assessment should ultimately be undertaken before commencing controlled normothermia in such patients.

### Auditing

1. ‘Time within target range’, ‘burden of fever’ and similar metrics can be considered as indicators of quality of temperature management.

‘Time within target range’ and ‘burden of fever’ were considered by the group to be appropriate metrics of quality temperature management. It was widely acknowledged that these metrics should be weighed by patient length of stay and/or duration of monitoring for appropriate statistical interpretation. The group was also careful to note that the administrative burden on physicians is already high and acknowledged the fact that some centres may not have access to electronic patient data management systems, so it was agreed that it was unrealistic for this group to issue prescriptive recommendations on auditing practices. In light of the high heterogeneity across centres [9], here the group were keen to clarify that wherever possible, documenting metrics such as ‘time within target range’ and ‘burden of fever’ may improve their ability to deliver data-driven service improvement and temperature control.

### Summary

This consensus review was undertaken to evaluate current evidence on the application of TTC in the management of severe TBI in a critical care setting, and to develop a set of practical recommendations to address identified gaps in current published evidence.

As highlighted by the SIBICC 2020 group, the gap between published evidence and management protocols is bridged by expert opinion [39]. The optimal method for the provision of high-quality TTC remains unknown, and barriers to its consistent implementation include the lack of evidence-based treatment protocols, knowledge deficiencies, limited access to equipment, lack of financial resources and staff workload. This document aims to address key practice gaps and optimise patient care through multimodal assessment following TBI.

## Strengths and limitations

The Delphi process has a number of strengths. Participants are able to reconsider their views in light of the evolving discussions, allowing for an element of reflection that isn’t regularly seen in other studies involving a single time point such as interviews or focus groups [40]. The element of anonymity offered to the panellists in the survey rounds avoids group conformity and promotes honesty, and the controlled and iterative discussions offer a flexible approach to gathering expert viewpoints on the set research questions. The Delphi method is an iterative process allowing the anonymous inclusion of a number of individuals across diverse locations and areas of expertise and avoiding dominance by any one individual. It uses a systematic progression of repeated rounds of voting and is an effective process for determining expert group consensus where there is little or no definitive evidence and where opinion is important [41, 42]. The modified Delphi approach used here combined the early flow of structured information and submission of anonymous responses with the (hybrid) face-to-face discussion and further voting to gain consensus (or establish lack thereof) and expert insight into usual practice regarding non-pharmacological TTC with an automated feedback-controlled device. As cited in existing literature however [13, 17], the Delphi process has limitations. The process is vulnerable to drop-outs and technical issues, with the online voting process during our meeting seeing some participants unable to cast their votes on a number of questions, leading to the need for a final anonymous survey round. The group opinions during the meeting may have been impacted by social bias, and the voices across the in-person and online participants may not have been equally heard, highlighting a potential need to ensure consistency in attendance in the same format in future panel meetings.

Our recommendations for the use of automated feedback-controlled TTC devices are based on expert consensus and theoretical benefits, such as precise temperature control and reduced temperature variability, which are thought to potentially improve outcomes in severe TBI management. We acknowledge the current evidence gap and strongly emphasise the need for rigorous research to evaluate the effectiveness of these devices, especially in diverse healthcare settings, including lower-income countries where resource limitations are critical. Future updates to these best-practice recommendations will incorporate emerging evidence to ensure relevance and applicability across different healthcare contexts, aiming for the highest standards of care within the constraints of available resources. While automated feedback-controlled TTC devices represent a significant advancement in the management of temperature in severe TBI patients, offering potential benefits in terms of precision and consistency, it is imperative to recognise the value and applicability of a wide range of temperature management approaches. These include both manual methods and simpler devices, which remain vital in many clinical settings around the world. Our guidelines advocate for the adaptation and implementation of TTC principles based on the specific resources, capabilities, and needs of each clinical setting.

This report has been developed by an expert panel comprised of specialists in neuro-critical care experienced in the management of severe TBI, therefore the recommendations focus on patients managed in a critical care environment. An individualised risk–benefit assessment should be undertaken for each domain to accommodate the high levels of heterogeneity seen across TBI patients, local practice settings, staff training and equipment availability [9].

## Conclusion

TTC is a therapy that has a role in ICP management and may reduce secondary injury and improve long-term neurological outcome for victims of TBI [9]. Appropriate methods for the implementation of TTC across widely heterogenous clinical settings and patient populations are relatively understudied, and due to a lack of consistent and high-quality evidence, remain largely unknown. Areas of consensus emerging from the Delphi process included TTC being recognised as an essential aspect of high-quality TBI care. Controlled normothermia (36.0–37.5 °C) was strongly recommended as a therapeutic option to be considered in Tier 1 and 2 of the SIBICC ICP management protocol. Temperature management targets should be individualised based on the perceived risk of secondary brain injury and fever aetiology.

### Supplementary Information

**Additional file 1**. Evaluation of five randomized controlled trials by the ESICM Methodology Group evaluates evulating cooling strategies against traditional interventions. The evaluation highlights methodological heterogeneities and evidential challenges.

**Additional file 2**. Delphi questionnaire: Round 1.

**Additional file 3**. Delphi questionnaire. Round 3.

**Additional file 4**. Systematic review of the literature on targeted temperature control in traumatic brain injury, covering clinical studies from 2013 to 2023.

## Data Availability

All data generated or analysed during this study are included in this article and its supplementary information files.

## Abbreviations

CPP: Cerebral perfusion pressure
CT: Computed tomography
EEG: Electroencephalography
ESAIC: European Society of Anaesthesiology and Intensive Care
ESICM: European Society of Intensive Care Medicine
Hb: Haemoglobin
ICP: Intracranial pressure
ICU: Intensive care unit
KPa: Kilopascal
NACCS: Neuro Anaesthesia and Critical Care Society
NaCl: Sodium chloride
NICU: Neuro-intensive care unit
NSAIDs: Nonsteroidal anti-inflammatory drugs
PaCO_2_: Arterial partial pressure of carbon dioxide
P_bt_O_2_: Brain tissue oxygenation
RCT: Randomised controlled trial
SIBICC: Seattle International Severe Traumatic Brain Injury Consensus Conference
SpO_2_: Arterial oxygen saturation
TBI: Traumatic brain injury
TTC: Targeted temperature control
