# Supplementary material for: Targeted temperature control following traumatic brain injury: ESICM/NACCS best practice consensus recommendations
Source: Crit Care. 2024 May 20;28:170. doi: 10.1186/s13054-024-04951-x (PMC11107011; doi:10.1186/s13054-024-04951-x)
Supplement: Supplementary file 1 — Additional file 1. Evaluation of five randomized controlled trials by the ESICM Methodology Group evaluates evulating cooling strategies against traditional interventions. The evaluation highlights methodological heterogeneities and evidential challenges. [file 13054_2024_4951_MOESM1_ESM.pdf]

Temperature management in traumatic brain injury (TTM TBI)

The ESICM Methodology Group

The ESICM Methodology Group is responsible to provide methodological expertise in ESICM guidelines or identify possible nominees for methodological support for the guidelines.
The group is also in charge of organizing activities concerning methodology for systematic reviews and guidelines.
The group also provides education and support to junior members, who have basic knowledge in the field of statistics and research methodology, involving them fully in the activities of the group.
Besides the Society research activities, the Methodology Group also supports other researches that have been endorsed by ESICM.

The ESICM Methodology Group approach is strongly based on the plausibility of the clinical hypothesis at the light of which the interpretation of a research and the statistical findings may change substantially.
The Methodology Group uses widespread tools as the Cochrane's ROB2 and the GRADE. However, assessment of evidence is integrated in those parts that are insufficiently covered by these tools. An example, is the "small trial bias" which is often overlooked in quality of evidence assessment. Our use of meta-analysis is consequently very parsimonious and is performed only when clinical and methodological criteria are met.

Dara Chean – Junior member of the ESICM Methodology Group
Daniele Poole – Chair of the ESICM Methodology Group

Temperature management in traumatic brain injury (TTM TBI)

Literature search and synthesis

The literature search provided 5 RCTs [1-5] dealing with temperature management in TBI.

RANDOMISED CONTROLLED TRIALS

Clinical heterogeneity

Patients

All the studies included patients with a GCS between 4 and 8 besides one that randomized patients with TBI higher than 20 mmHg regardless of GCS [1].

Interventions

Controls

In the Eurotherm3235 [1], the control group received osmotic therapy that was not allowed in the intervention arm. However, the effectiveness of mannitol in reducing ICP has strong pharmacodynamic and pathophysiological basis and is supported by some evidence [6]. Thus, there is a high risk that we are comparing the intervention with an unfair outcome.

Selected outcomes (and corresponding Forest plots)

The trials considered good outcome at 6 months, either using the GOSe 5 to 6 [1, 2], or GOS 4 and 5 [3-5].

Individual quality of evidence and methodological heterogeneity

The Eurotherm3235 [1] was of high quality according to the ROB2 and the GRADE tools, however, the use of an unfair comparison hampered the value in terms of evidence of this study. On the other hand, in the POLAR trial [2] a high number of patients did not receive the planned treatment, prevalently in the experimental arm (table 1). We considered this study at high risk of bias relying on the ROB2, although no concerns were raised when applying the GRADE tool for individual studies (table 2).

The trial by Qui [5], although not raising concerns according to the ROB2 e and GRADE assessment, was at high risk of “small trial bias”. Indeed, an exaggerated protective and

statistically significant effect was found with only 86 patients randomized. In our evaluation this study provided only very low evidence.

We regarded evidence provided by the trials by Hui and Maekawa [3, 4] of high quality, instead. However, both studies assumed an exaggerated protective effect that biased the sample size calculation, affecting the possibility of having a positive trial. Thus, false negative findings could have been provided by both. In the end although evidence was rated high according to the ROB2 and GRADE, findings from these two studies should be regarded as inconclusive.

Overall quality of evidence and statistical heterogeneity

The results of the trials are reported in table 3 and figure 1. They were considered too heterogenous in clinical and methodological terms to be combined meta-analytically and no overall evidence could be provided. In general, we could conclude that the subject was insufficiently addressed and would need further investigations.

External validity

All the trials randomized a small number of patients per center and year (table 3). We cannot be sure that the selection occurred at random and thus there could be an issue of selection bias.

Temperature management in traumatic brain injury (TTM TBI)

	Andrews - NEJM 2015		Cooper - JAMA 2018		Hui - eCM 2021		Maekawa - JNT 2015		Qiu - BI 2022	
**Study arm**	Experimental	Control	Experimental	Control	Experimental	Control	Experimental	Control	Experimental	Control
**Patients**	ICU patients with TBI and an intracranial pressure of more than 20 mm Hg for at least 5 minutes after stage 1 treatments		Emergency department patients with TBI and GCS < 9, excluding those with GCS 3 and unreactive pupils		Patients with closed head injury, presenting within 24 hours, with a Glasgow Coma Scale of 4 to 8 after resuscitation, initial ICP > 24 mm Hg		TBI with GCS score between 4 and 8		TBI with GCS score between 4 and 8	
**Intervention and controls**	Hypothermia 32-35° C to obtain ICP of 20 mmHg or less	Standard treatment plus mannitol	Hypothermia 33°C	Standard treatment	Hypothermia 34-35° C	Standard treatment	Hypothermia 32-34°C	Standard treatment	Prehospital hypothermia 33-35°C	Standard treatment
**N patients at randomization (denominator for statistics)**	191	189	260	240	156	146	98	50	43	43
**N patients available for outcome analysis**	191	189	240	226	138	133	94	48	43	43
**N/% of patients lost for outcome analysis**	No patients lost	No patients lost	20 (7.7%) patients lost	14 (5.8%) patients lost	18 (11.5%) patients lost	13 (8.9%) patients lost	4 (4.1%) patients lost	2 (4%) patients lost	No patients lost	No patients lost
**Age Mean (SD)**	37.4 (15.4)	36.7 (14.9)	35 (13.5)	34.1 (13.4)	44.7 (13)	49.1 (12.82)	39 (19)	39 (18)	42.3 (1.8)	40.6 (7.6)
**Females n (%)**	38 (19.9)	28 (14.8)	53 (20.4)	46 (19.2)	29 (18.6)	34 (23.3)	29 (29.6)	16 (32)	20 (46.5)	22 (51.2)
**Protocol violations n (%)**	1 (0.5)	0 (0)	108 (41.5)	65 (27.1)	NA (NA)	NA (NA)	61 (62.2)	4 (8)	NA (NA)	NA (NA)
**Missing outcomes n (%)**	4 (2.1)	3 (1.6)	20 (7.7)	14 (5.8)	18 (11.5)	13 (8.9)	4 (4.1)	2 (4)	0 (0)	0 (0)
**Pupillary response (reacting) n (%)**	144 (75.4)	143 (75.7)	220 (84.6)	202 (84.2)	86 (55.1)	77 (52.7)	47 (48)	23 (46)	NA (NA)	NA (NA)
**ICP mmHg Mean (SD)**	25.2 (13.2)	25.5 (13.5)	NA (NA)	A (NNA)	106 (67.9)	104 (71.2)	6 (6.1)	5 (10)	NA (NA)	NA (NA)
**Decompressive craniectomy n (%)**	27 (14.1)	27 (14.3)	NA (NA)	NA (NA)	106 (67.9)	104 (71.2)	6 (6.1)	5 (10)	NA (NA)	NA (NA)
**GCS**	NA	NA	6 (4-7)*	6 (4-7)*	NA	NA	5.8 (1.4)	5.9 (1.3)	NA	NA
**Centers included**	47		13		14		NA		3	
**n pts/centre/year**	2		5		4		NA		5	

Table 1: main characteristics of the trials that were reviewed.

Temperature management in traumatic brain injury (TTM TBI)

ROB2	Bias arising from the randomization process	Bias due to deviations from intended interventions	Bias due to missing outcome data	Bias in measurement of the outcome	Bias in selection of the reported result	Overall risk of bias
Andrews NEJM-2015	+	+	+	+	+	+
Cooper JAMA-2018	−	×	+	+	+	×
Hui eCM-2021	−	−	−	+	+	−
Maekawa JNT-2015	+	−	+	+	+	−
Qiu BI-2022	−	+	+	+	+	−

Table 2: quality of evidence assessment according to the ROB2

Temperature management in traumatic brain injury (TTM TBI)

			Intervention				Control					
author	journal	year	N events	% events	N non-events	tt	N events	% events	N non-events	ct	Delta	reported p value
Andrews	NEJM	2015	49	25.7	142	191	69	36.5	120	189	-10.9	0.03
Cooper	JAMA	2018	117	48.8	123	240	111	49.1	115	226	-0.4	0.94
Hui	eCM	2021	81	58.7	57	138	64	48.1	69	133	10.6	0.081
Maekawa	JNT	2015	44	46.8	50	94	25	52.1	23	48	-5.3	0.597
Qiu	BI	2022	28	65.1	15	43	16	37.2	27	43	27.9	<0.05

Table 3: findings of the trials that were reviewed

Temperature management in traumatic brain injury (TTM TBI)

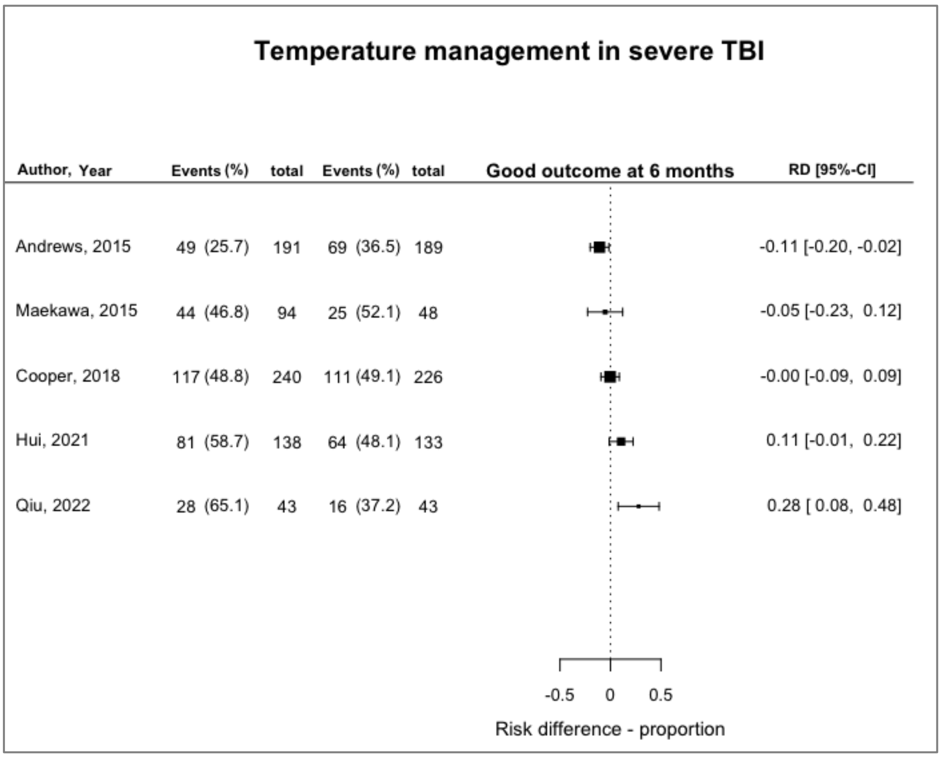

Figure 1: outcome of the trials that were reviewed

Temperature management in traumatic brain injury (TTM TBI)

Hypothemia in severe TBI

9/17/2023

SINGLE RCT QUALITY ASSESSMENT	Question: Does hypothermia as a strategy to control intracranial hypertension improve neurologic outcomes in patients with traumatic brain injury?		
RCT 1	Parallel	Superiority	III
Andrews	NEJM	2015	Centers included
Patients	ICU patients with TBI and an intracranial pressure of more than 20 mm Hg for at le		47
Treatment	Hypothermia 32-35° C to obta	Fragility index for p values < 0.05, Based on the z test	
Control	Standard treatment plus mar	4	
Outcome	GOS-E 5 to 8 at 6 months	Outcome reported in the trial registration	
		Outcome quality: Clinically important	Undesirable effects
	Number of patients	n (%)	Adverse events %
Treatment	191	49 (25.7)	17.3
Control	189	69 (36.5) [Expected rate: NA]	5.3
Total	380	118 (31.1)	11.3 (p < 0.0001)
delta -10.9 (95%-CI -19.9 to -1.5)		NNTB 9 (95%-CI NNTB 5 to NNTB 65)	
Sample size calculation not based on this outcome	p for effect size 0.03 (calculated 0.022)	Power for the observed delta: 0.63	Power by design: 0.8
Planned sample size:	Sample size needed for observed delta: 546		
Calculated sample size for predicted delta: not computable		GRADE CRITERIA	
Downgrade (GRADE AND ROB2)		Lack of allocation concealment	No
		Lack of Blinding	Yes
		Selective outcome reporting bias	No
		Incomplete accounting of patients and outcome events	No
		Stopping early for benefit/using unvalidated outcome measures	No
		Risk of bias of the single RCT	No
		ROB2 CRITERIA	
		Bias arising from the randomization process	Low risk
		Bias due to deviations from intended interventions	Low risk
		Bias due to missing outcome data	Low risk
		Bias in measurement of the outcome	Low risk
		Bias in selection of the reported result	Low risk
		Overall risk of bias	Low risk
		SMALL TRIAL BIAS	
		No small-trial bias issues	No risk of bias
Comments		GRADE: Lack of Blinding: Outcome measurment was performed blindly, lack of blinding should not affect the mortality; Stopping early for benefit/using unvalidated outcome measures: Stopped early for concerns of harm or probable futility; Methodological and statistical quality: Mannitol therapy was allowded only in the control arm, creating an unfari comparison; ROB2:Overall risk of bias: Low risk; Small trial bias: No risk of bias.	
Methodological and statistical quality	Statistical reporting (CONSORT)	**Overall risk of bias**	External validity issues
Low	Adequate	GRADE: No; ROB2: Low risk; Small Trial Bias: No risk of bias	Few patients randomized per centre

Temperature management in traumatic brain injury (TTM TBI)

Hypothemia in severe TBI 9/17/2023

**SINGLE RCT QUALITY ASSESSMENT**	Question: Does prophylactic early hypothermia during at least 72 hours improve neurologic outcomes in patients with traumatic brain injury?		
RCT 3	Parallel	Superiority	III
Cooper	JAMA	2018	Centers included
Patients	Emergency department patients with TBI and GCS < 9, excluding those with GCS 3		13
Treatment	Hypothermia (33°C)	Fragility index for p values < 0.05, Based on the z test	
Control	Standard treatment	Not indicated	
Outcome	GOS-E 5 to 8 at 6 months	Outcome reported in the trial registration	
		Outcome quality: Clinically important	Undesirable effects
	Number of patients	n (%)	Adverse effects %
Treatment	240	117 (48.8)	NR
Control	226	111 (49.1) [Expected rate: 50 %]	NR
Total	466	228 (48.9)	NR ()
delta -0.4 (95%-CI -9.4 to 8.6)		NNTB 274 (95%-CI NNTB 11 to ∞ to NNTH 12)	
Expected delta: 15	p for effect size 0.94 (calculated 0.938)	Power for the observed delta: 0.03	Power by design: 0.8
Planned sample size: 364	Sample size needed for observed delta: 571363		
Calculated sample size for predicted delta: 333		**GRADE CRITERIA**	
Downgrade (GRADE AND ROB2)		Lack of allocation concealment	Maybe
		Lack of Blinding	Yes
		Selective outcome reporting bias	No
		Incomplete accounting of patients and outcome events	No
		Stopping early for benefit/using unvalidated outcome measures	No
		Risk of bias of the single RCT	No
		**ROB2 CRITERIA**	
		Bias arising from the randomization process	Some concerns
		Bias due to deviations from intended interventions	High risk
		Bias due to missing outcome data	Low risk
		Bias in measurement of the outcome	Low risk
		Bias in selection of the reported result	Low risk
		Overall risk of bias	High risk
		**SMALL TRIAL BIAS**	
		No small-trial bias issues	No risk of bias
Comments		GRADE: Lack of allocation concealment: Some concerns for the use of sealed opaque envelopes; Lack of Blinding: It should not affect the outcome; Methodological and statistical quality: There was a high number of protocol violations particularly in the intervention arm; ROB2:Bias arising from the randomization process: Some concerns for the use of sealed opaque envelopes; Bias due to deviations from intended interventions: A high number of patients in both groups did not receive the planned treatment; Overall risk of bias: High risk; Small trial bias: No risk of bias.	
Methodological and statistical quality	Statistical reporting (CONSORT)	**GRADE and ROB2 criteria**	External validity issues
High	Adequate	GRADE: No; ROB2: High risk; Small Trial Bias: No risk of bias	Few patients randomized per centre

Temperature management in traumatic brain injury (TTM TBI)

Hypothemia in severe TBI

9/17/2023

SINGLE RCT QUALITY ASSESSMENT	Question: Does hypothermia (34 to 35°C) during 5 days after traumatic brain injury with intracranial hypertension improve neurologic outcome at 6 month?		
RCT 4	Parallel	Superiority	III
Hui	eCM	2021	Centers included
Patients available for analy	Patients with closed head injury, presenting within 24 hours, with a Glasgow Coma		14
Treatment	Hypothermia 34-35° C, for 5 d	Fragility index for p values < 0.05, Based on the z test	
Control	Standard treatment	Not indicated	
Outcome	Favorable GOS (4-5) at 6 mo	Outcome reported in the trial registration	
		Outcome quality: Clinically important	Undesirable effects
	Number of patients	n (%)	Adverse effects %
Treatment	138	81 (58.7)	NR
Control	133	64 (48.1) [Expected rate: 27 %]	NR
Total	271	145 (53.5)	NR ()
delta 10.6 (95%-CI -1.3 to 22)		NNTH 9 (95%-CI NNTB 78 to ∞ to NNTH 5)	
Expected delta: 16	p for effect size 0.081 (calculated 0.08)	Power for the observed delta: 0.42	Power by design: 0.8
Planned sample size: 272	Sample size needed for observed delta: 670		
Calculated sample size for predicted delta: 271		**GRADE CRITERIA**	
Downgrade (GRADE AND ROB2)		Lack of allocation concealment	Maybe
		Lack of Blinding	Yes
		Selective outcome reporting bias	No
		Incomplete accounting of patients and outcome events	No
		Stopping early for benefit/using unvalidated outcome measures	No
		Risk of bias of the single RCT	No
		**ROB2 CRITERIA**	
		Bias arising from the randomization process	Some concerns
		Bias due to deviations from intended interventions	Some concerns
		Bias due to missing outcome data	Some concerns
		Bias in measurement of the outcome	Low risk
		Bias in selection of the reported result	Low risk
		Overall risk of bias	Some concerns
		**SMALL TRIAL BIAS**	
		No small-trial bias issues	No risk of bias
Comments		GRADE: Lack of allocation concealment: Some concerns for the use of sealed opaque envelopes; Lack of Blinding: It should not affect the outcome because assessment of the outcome was performed blindigly; Methodological and statistical quality: Exagerated outcome reduction used for sample size computation; ROB2:Bias arising from the randomization process: Some concerns for the use of sealed opaque envelopes; Bias due to deviations from intended interventions: 88% of the patients reached the targeted temperature in the intervention arm; Bias due to missing outcome data: 10% of patients lost to follow-up, balanced between the study arms with a mean temperature of 35.6° C; Overall risk of bias: Some concerns; Small trial bias: No risk of bias.	
Methodological and statistical quality	Statistical reporting (CONSORT)	**Overall risk of bias**	External validity issues
Low	Adequate	GRADE: No; ROB2: Some concerns; Small Trial Bias: No risk of bias	Few patients randomized per centre

Temperature management in traumatic brain injury (TTM TBI)

Hypothemia in severe TBI

9/17/2023

SINGLE RCT QUALITY ASSESSMENT	Question: Does hypothermia (32 to 34°C) during at least 3 days after traumatic brain injury improve neurologic outcome at 6 month?		
RCT 5	Parallel	Superiority	
Maekawa	JNT	2015	Centers included
Patients	TBI with GCS score between 4 and 8		NA
Treatment	Hypothermia 32-34°C	Fragility index for p values < 0.05, Based on the z test	
Control	Standard treatment	Not indicated	
Outcome	Favorable GOS (4-5) at 6 mo	Outcome reported in the trial registration	
		Outcome quality: Clinically important	Undesirable effects
	Number of patients	n (%)	Adverse events %
Treatment	94	44 (46.8)	18.1
Control	48	25 (52.1) [Expected rate: 31 %]	2.1
Total	142	69 (48.6)	12.7 (p < 0.0001)
delta -5.3 (95%-CI -21.9 to 11.7)		NNTB 19 (95%-CI NNTB 5 to ∞ to NNTH 9)	
Expected delta: 20	p for effect size 0.597 (calculated 0.552)	Power for the observed delta: 0.09	Power by design: 0.9
Planned sample size: 300	Sample size needed for observed delta: 2931		
Calculated sample size for predicted delta: 244 (2(intervention):1(control) randomization)		**GRADE CRITERIA**	
Downgrade (GRADE AND ROB2)		Lack of allocation concealment	No
		Lack of Blinding	Yes
		Selective outcome reporting bias	No
		Incomplete accounting of patients and outcome events	No
		Stopping early for benefit/using unvalidated outcome measures	No
		Risk of bias of the single RCT	No
		**ROB2 CRITERIA**	
		Bias arising from the randomization process	Low risk
		Bias due to deviations from intended interventions	Some concerns
		Bias due to missing outcome data	Low risk
		Bias in measurement of the outcome	Low risk
		Bias in selection of the reported result	Low risk
		Overall risk of bias	Some concerns
		**SMALL TRIAL BIAS**	
		No small-trial bias issues	No risk of bias
Comments		GRADE: Lack of Blinding: It should not affect the outcome because assessment of the outcome was performed blindigly; Methodological and statistical quality: Exagerated outcome reduction used for sample size computation; ROB2:Overall risk of bias: Some concerns; Small trial bias: No risk of bias.	
Methodological and statistical quality	Statistical reporting (CONSORT)	**Overall risk of bias**	External validity issues
Low	Adequate	GRADE: No; ROB2: Some concerns; Small Trial Bias: No risk of bias	

Temperature management in traumatic brain injury (TTM TBI)

Hypothemia in severe TBI

9/17/2023

**SINGLE RCT QUALITY ASSESSMENT**	Question: Does hypothermia (33 to 35°C) started from pre hospital care improve neurological outcome compared to hypothermia started on ICU admission in traumatic brain injury?		
RCT 6	Parallel	Superiority	
Qiu	BI	2022	Centers included
Patients available for analysi	TBI with GCS score between 4 and 8		3
Treatment	Prehospital hypothermia 33-3	Fragility index for p values < 0.05, Based on the z test	
Control	Standard treatment	12	
Outcome	Favorable GOS (4-5) at 6 mo	Unregistered trial	
		Outcome quality: Clinically important	Undesirable effects
	Number of patients	n (%)	Adverse effects %
Treatment	43	28 (65.1)	NR
Control	43	16 (37.2) [Expected rate: NA]	NR
Total	86	44 (51.2)	NR ()
delta 27.9 (95%-CI 6.8 to 45.8)		NNTH 4 (95%-CI NNTH 15 to NNTH 2)	
Expected delta not reported	p for effect size <0.05 (calculated 0.008)	Power for the observed delta: 0.77	Power by design: Not reported
No sample size calculation	Sample size needed for observed delta: 90		
Calculated sample size for predicted delta: not computable		**GRADE CRITERIA**	
Downgrade (GRADE AND ROB2)		Lack of allocation concealment	Maybe
		Lack of Blinding	Yes
		Selective outcome reporting bias	No
		Incomplete accounting of patients and outcome events	No
		Stopping early for benefit/using unvalidated outcome measures	Not reported
		Risk of bias of the single RCT	No
		**ROB2 CRITERIA**	
		Bias arising from the randomization process	Some concerns
		Bias due to deviations from intended interventions	Low risk
		Bias due to missing outcome data	Low risk
		Bias in measurement of the outcome	Low risk
		Bias in selection of the reported result	Low risk
		Overall risk of bias	Some concerns
		**SMALL TRIAL BIAS**	
		Warning for small-trial bias with exaggerated effect	High risk of bias
Comments		GRADE: Lack of allocation concealment: Some concerns for the use of sealed opaque envelopes; ROB2:Bias arising from the randomization process: Some concerns for the use of sealed opaque envelopes; Overall risk of bias: Some concerns; Small trial bias: High risk of bias.	
Methodological and statistical quality	Statistical reporting (CONSORT)	**Overall risk of bias**	External validity issues
Low	Partial and not sufficient for quality assessment	GRADE: No; ROB2: Some concerns; Small Trial Bias: High risk of bias	Surgery 90-95%

Temperature management in traumatic brain injury (TTM TBI)

References

1. Andrews PJ, Sinclair HL, Rodriguez A, Harris BA, Battison CG, Rhodes JK, Murray GD, Eurotherm Trial C, (2015) Hypothermia for Intracranial Hypertension after Traumatic Brain Injury. N Engl J Med 373: 2403-2412
2. Cooper DJ, Nichol AD, Bailey M, Bernard S, Cameron PA, Pili-Floury S, Forbes A, Gantner D, Higgins AM, Huet O, et al., (2018) Effect of Early Sustained Prophylactic Hypothermia on Neurologic Outcomes Among Patients With Severe Traumatic Brain Injury: The POLAR Randomized Clinical Trial. JAMA 320: 2211-2220
3. Hui J, Feng J, Tu Y, Zhang W, Zhong C, Liu M, Wang Y, Long L, Chen L, Liu J, et al., (2021) Safety and efficacy of long-term mild hypothermia for severe traumatic brain injury with refractory intracranial hypertension (LTH-1): A multicenter randomized controlled trial. EClinicalMedicine 32: 100732
4. Maekawa T, Yamashita S, Nagao S, Hayashi N, Ohashi Y, Brain-Hypothermia Study G, (2015) Prolonged mild therapeutic hypothermia versus fever control with tight hemodynamic monitoring and slow rewarming in patients with severe traumatic brain injury: a randomized controlled trial. J Neurotrauma 32: 422-429
5. Qiu W, Chen M, Wang X, Qiu W, Chen M, Wang X, (2022) Pre-hospital mild therapeutic hypothermia for patients with severe traumatic brain injury. Brain Inj 36: 72-76
6. Poole D, Citerio G, Helbok R, Ichai C, Meyfroidt G, Oddo M, Payen JF, Stocchetti N, (2020) Evidence for Mannitol as an Effective Agent Against Intracranial Hypertension: An Individual Patient Data Meta-analysis. Neurocrit Care 32: 252-261
