# Supplementary material for: Targeted temperature control following traumatic brain injury: ESICM/NACCS best practice consensus recommendations
Source: Crit Care. 2024 May 20;28:170. doi: 10.1186/s13054-024-04951-x (PMC11107011; doi:10.1186/s13054-024-04951-x)
Supplement: Supplementary file 2 — Additional file 2. Delphi questionnaire: Round 1. [file 13054_2024_4951_MOESM2_ESM.pdf]

TTM for TBI management consensus meeting - Delphi questions

1. Introduction

Scope of work:
The following questions and recommendations apply specifically to patients with severe traumatic brain injury (TBI), defined as a Glasgow Coma Scale (GCS) score of 3–8, and to patients with moderate TBI that subsequently deteriorate and require admission to ICU for intracranial pressure (ICP) management.

Working definitions:
Various definitions of normothermia and hypothermia ranges are provided in the literature depending on clinical contexts. For the purpose of this work, the following pragmatic definitions are proposed:

Normothermia: core body temperature 36.0°C to 37.5°C
Mild Hypothermia: core body temperature ranging from 34.0°C to 36.0°C
Moderate Hypothermia: 34.0°C to 32.0°C
Extreme hypothermia: core body temperature 32.0°C to 28.0°C *
Deep hypothermia: core body temperature below 28.0°C

***We pragmatically introduce the term "extreme hypothermia" to define the temperature range between 28.0°C and 32.0°C. This designation underscores a range where temperatures notably deviate from the target temperature ranges established in the existing literature for severe TBI (i.e., 32.0–35.0°C). The term "deep hypothermia" is widely accepted for core temperatures below 28.0°C.**

Definition of severe TBI: Teasdale G, Jennett B. Assessment of coma and impaired consciousness. A practical scale. Lancet. 1974 Jul 13;2(7872):81–4.

1. Full name *

2. Email Address *

3. Telephone number *

2. Pathophysiology

4. Precise temperature control and management is an essential aspect of high-quality care in patients with severe traumatic brain injury (TBI) *

- [ ] True
- [ ] False

Comments:

5. Uncontrolled fever (neurogenic or secondary to inflammation or infection) can precipitate secondary brain injury in patients with severe TBI
*****

- [ ] True
- [ ] False

Comments:

6. Abnormally elevated temperature can increase the risk of seizures in patients with severe TBI *

- [ ] True
- [ ] False

Comments:

7. Abnormally elevated temperature increases the risk of intracranial hypertension in patients with severe TBI *

- [ ] True
- [ ] False

Comments:

8. Abnormally elevated temperature increases the risk of brain herniation in patients with severe TBI *

- [ ] True
- [ ] False

Comments:

3. Monitoring

9. Continuous temperature monitoring is preferable over intermittent temperature measurements in patients with severe TBI *

- [ ] True

☐ False

Comments:

10. Monitoring core temperature (e.g., bladder, oesophageal, brain) is strongly recommended over measuring or monitoring superficial temperature (e.g., skin, tympanic) in severe TBI *

☐ True

☐ False

Comments:

11. Monitoring target organ (i.e. brain) temperature is desirable and recommended over alternative core temperature monitoring options when the insertion of intraparenchymal brain probes is indicated and the technology is available *

☐ True

☐ False

Comments:

12. When brain temperature monitoring is not immediately available, alternative sources of core temperature (oesophageal, bladder, intravascular) are acceptable *

☐ True

☐ False

Comments:

13. When brain temperature monitoring is available, it is advisable to simultaneously assess brain temperature and core temperature in order to improve patient safety (i.e. reduce the risk of thermometer failure) *

☐ True

☐ False

Comments:

4. ICP

14. Temperature control is a key component of intracranial pressure (ICP) management in severe TBI cases *

☐ True

☐ False

Comments:

15. Controlled normothermia (i.e. target core temperature 36.0°C–37.5°C) should be considered in cases where first-line treatments (e.g., sedation, osmotherapy, optimised ventilation) have failed to control ICP *

- [ ] True
- [ ] False
- [ ] Other (please specify):

Comments:

16. Mild to moderate hypothermia (i.e. target core temperature ≤36.0°C) should be considered in cases where first-line treatments (e.g., sedation, osmotherapy, optimised ventilation) have failed to control ICP *

- [ ] True
- [ ] False

Comments:

17. If hypothermia is considered in cases where first-line treatments have failed to control ICP, target temperature should be managed as close to physiological temperature as possible (i.e. starting from mild hypothermia and progressively cooling patients to moderate hypothermia) *

- [ ] True
- [ ] False

Comments:

18. In cases where patients are at impending risk of brain herniation (i.e., obliterated basal cisterns, refractory intracranial hypertension >25 mmHg, abnormal pupillary reaction), therapeutic hypothermia should be considered as a temporising strategy, and it should be induced rapidly *

- [ ] True
- [ ] False

Comments:

19. In a patient at risk of impending herniation awaiting surgical evacuation or decompression, the lowest target core temperature that a short-term temporising strategy should be implemented at is: *

- [ ] 32.0°C
- [ ] 33.0°C
- [ ] 34.0°C
- [ ] 35.0°C
- [ ] Other (please specify):

Comments:

20. In a patient with exhausted intracranial volume buffering reserve and labile ICP with occasional spikes >25 mmHg, the lowest target core temperature that a medium term ICP-control strategy should be implemented at is: *

- [ ] 32.0°C
- [ ] 33.0°C

- [ ] 34.0°C
- [ ] 35.0°C
- [ ] Other (please specify):

Comments:

21. Before considering decompressive craniectomy, moderate hypothermia should be attempted, with a target core temperature no lower than: *

- [ ] 32.0°C
- [ ] 33.0°C
- [ ] 34.0°C
- [ ] 35.0°C
- [ ] Therapeutic hypothermia not an option
- [ ] Other (please specify):

Comments:

22. Before considering barbiturate burst suppression, moderate hypothermia should be attempted, with a target core temperature no lower than: *

- [ ] 32.0°C
- [ ] 33.0°C
- [ ] 34.0°C
- [ ] 35.0°C
- [ ] Therapeutic hypothermia not an option

☐ Other (please specify):

Comments:

5. Fever

23. Neurogenic fever (core temperature >37.5°C driven by neurological dysregulation in the absence of sepsis or clinically significant inflammatory process) is relatively common in traumatic brain injury cases, and it should be promptly detected and treated (i.e., with controlled normothermia targeting 36.0°C to 37.5°C). *

☐ True

☐ False

☐ Other (please specify):

Comments:

24. Controlled normothermia should be considered when pyrexia is secondary to sepsis or inflammatory processes, and when the patient is perceived to be at risk of secondary brain injury, especially in the acute phase of TBI *

☐ True

☐ False

☐ Other (please specify):

Comments:

25. When fever is detected following severe TBI in cases perceived to be at risk of secondary brain injury, target temperature management should be initiated with a target core temperature range of 36.0°C to 37.5°C (i.e. controlled normothermia) *

- [ ] True
- [ ] False
- [ ] Other (please specify):

Comments:

26. In patients with severe TBI who are sedated and ventilated for ICP management, controlled normothermia should be initiated: *

- [ ] Preventively
- [ ] Reactively when core temperature exceeds 36.5°C
- [ ] Reactively when core temperature exceeds 37.0°C
- [ ] Reactively when core temperature exceeds 37.5°C
- [ ] Reactively when core temperature exceeds 38.0°C
- [ ] Other (please specify):

Comments:

27. When neurogenic fever is detected in TBI cases, controlled normothermia should be continued for as long as the brain remains at risk of secondary brain damage (i.e. labile ICP, temporal contusions, obliterated basal cisterns, midline shift / subfalcine herniation and other signs of exhausted intracranial volume-buffering reserve or risk of seizures) *

- [ ] True
- [ ] False
- [ ] Other (please specify):

Comments:

6. TTM induction

28. Rapid induction of hypothermia in traumatic brain injury cases can be achieved with a combination of automated temperature control devices and, in selected cases, intravenous infusion of ice-cold solutions *

- [ ] True
- [ ] False
- [ ] Other (please specify):

Comments:

29. It is advisable that neurotrauma ICUs should stock readily available NaCl solutions of different concentrations stored at ice-cold temperature for the management of intracranial hypertension crises *

- [ ] True

☐ False

☐ Other (please specify):

Comments:

7. TTM maintenance

30. An automated feedback-controlled TTM device that enables precise temperature control is desirable for the initiation of TTM and maintenance at target temperature in patients with severe TBI *

☐ True

☐ False

☐ Other (please specify):

Comments:

31. The maximum temperature variation that a patient should experience during normothermia is less than or equal to +/- 0.5°C per hour and ≤1°C per 24-h period *

☐ True

☐ False

☐ Other (please specify):

Comments:

32. When mild or moderate hypothermia is indicated, treatment should be continued for as long as the brain remains at risk of secondary brain damage (i.e. labile ICP, obliterated basal cisterns, midline shift / subfalcine herniation and other signs of exhausted intracranial volume-buffering reserve) *

- [ ] True
- [ ] False
- [ ] Other (please specify):

Comments:

8. TTM rewarming

33. Obtaining an interval scan and/or an alternative assessment of intracranial volume buffering reserve (i.e. intracranial compliance, ICP pulse amplitude, etc.) is recommended before considering rewarming *

- [ ] True
- [ ] False
- [ ] Other (please specify):

Comments:

34. Rewarming should be controlled by an automated feedback-controlled TTM device and set at a rate of ≤1°C per 24-h period *

- [ ] True
- [ ] False
- [ ] Other (please specify):

Comments:

35. Rebound hyperthermia should be prevented whenever possible or promptly treated in cases when the brain is perceived to be at risk of secondary brain injury *

- [ ] True
- [ ] False
- [ ] Other (please specify):

Comments:

9. Shivering

36. It is important to assess, document and manage shivering in severe TBI patients *

- [ ] True
- [ ] False
- [ ] Other (please specify):

Comments:

37. Whenever ICP is labile and shivering is detected, neuromuscular blockers should be considered *

- [ ] True
- [ ] False
- [ ] Other (please specify):

Comments:

38. In self-ventilating patients in the subacute phase of severe TBI, an individualised risk-benefit assessment should be undertaken regarding the indications of controlled normothermia. *

- [ ] True
- [ ] False
- [ ] Other (please specify):

Comments:

39. Permissive hyperthermia should be considered in cases where risk of secondary brain injury resulting from pyrexia is thought to be low, and when shivering cannot be controlled with first line treatments such as NSAIDs, opiates, magnesium or counter warming *

- [ ] True
- [ ] False
- [ ] Other (please specify):

Comments:

10. Auditing

40. The quality of temperature management should be prospectively audited with metrics such a time within target range *

- [ ] True
- [ ] False
- [ ] Other (please specify):

Comments:
