# Supplementary material for: Targeted temperature control following traumatic brain injury: ESICM/NACCS best practice consensus recommendations
Source: Crit Care. 2024 May 20;28:170. doi: 10.1186/s13054-024-04951-x (PMC11107011; doi:10.1186/s13054-024-04951-x)
Supplement: Supplementary file 3 — Additional file 3. Delphi questionnaire. Round 3. [file 13054_2024_4951_MOESM3_ESM.pdf]

TTM for TBI management consensus: Round 3 Delphi questions

1. Introduction

Scope of work:
As in round 1 and 2, the following questions and recommendations apply specifically to patients with severe traumatic brain injury (TBI), defined as a Glasgow Coma Scale (GCS) score of 3–8, and to patients with moderate TBI that subsequently deteriorate and require admission to ICU for intracranial pressure (ICP) management. This round 3 includes the questions that were updated during the round 2 meeting on Tuesday 10 October 2023, that we would like you to revote on to achieve final consensus.

Working definitions:
Various definitions of normothermia and hypothermia ranges are provided in the literature depending on clinical contexts. For the purpose of this work, the following pragmatic definitions are proposed:

Normothermia: core body temperature 36.0°C to 37.5°C
Mild Hypothermia: core body temperature ranging from 34.0°C to 36.0°C
Moderate Hypothermia: 34.0°C to 32.0°C
Extreme hypothermia: core body temperature 32.0°C to 28.0°C *
Deep hypothermia: core body temperature below 28.0°C

***We pragmatically introduce the term "extreme hypothermia" to define the temperature range between 28.0°C and 32.0°C. This designation underscores a range where temperatures notably deviate from the target temperature ranges established in the existing literature for severe TBI (i.e., 32.0–35.0°C). The term "deep hypothermia" is widely accepted for core temperatures below 28.0°C.**

Definition of severe TBI: Teasdale G, Jennett B. Assessment of coma and impaired consciousness. A practical scale. Lancet. 1974 Jul 13;2(7872):81–4.

1. Full name *

2. Email Address *

3. Telephone number *

2. Pathophysiology

4. Temperature measurement and control is an essential aspect of high-quality care in patients with severe traumatic brain injury (TBI). *

- [ ] True
- [ ] False

Comments:

5. Uncontrolled fever (neurogenic or secondary to inflammation or infection) can precipitate secondary brain injury in patients with severe TBI
*****

- [ ] True
- [ ] False

Comments:

6. Fever control is recommended in patients with severe TBI who have seizures or are perceived to be at high risk of seizures. *

- [ ] True
- [ ] False

Comments:

7. Fever increases the risk of intracranial hypertension in patients with severe TBI *

- [ ] True
- [ ] False

Comments:

8. In patients with impending cerebral herniation, temperature control is essential. *

- [ ] True
- [ ] False

Comments:

3. Monitoring

9. Continuous temperature monitoring is preferable over intermittent temperature measurements in patients with severe TBI *

- [ ] True

☐ False

Comments:

10. Monitoring core temperature (e.g., bladder, oesophageal, brain) is strongly recommended over measuring or monitoring superficial temperature (e.g., skin, tympanic) in severe TBI *

☐ True

☐ False

Comments:

11. Monitoring brain temperature is recommended in addition to monitoring core systemic temperature as a therapeutic target *

☐ True

☐ False

Comments:

12. When brain temperature monitoring is not immediately available, alternative sources of core temperature (oesophageal, bladder, intravascular) are acceptable *

☐ True

☐ False

Comments:

13. When brain temperature monitoring is in place, it is advisable to also assess core temperature *

- [ ] True
- [ ] False

Comments:

4. ICP

14. Temperature control is a key component of intracranial pressure (ICP) management in severe TBI cases *

- [ ] True
- [ ] False

Comments:

15. Controlled normothermia (i.e. target core temperature 36.0°C–37.5°C) should be considered in addition to Tier 1 and Tier 2 treatments (SIBICC 2019: Tier 0: prevent fever; Tier 3: mild hypothermia using active cooling measures) *

- [ ] True
- [ ] False
- [ ] Other (please specify):

Comments:

16. Therapeutic hypothermia (i.e. target core temperature ≤36.0°C) should be considered in cases where tier 1 and 2 treatments (as per SIBICC guidance) have failed to control ICP *

- [ ] True
- [ ] False

Comments:

17. If hypothermia is considered to control ICP, target temperature should be managed as close to physiological temperature as possible *

- [ ] True
- [ ] False

Comments:

18. In patients with impending brain herniation, therapeutic hypothermia should be considered as a temporising strategy, and should be induced rapidly *

- [ ] True
- [ ] False

Comments:

19. In a patient with impending herniation awaiting surgical evacuation or decompression, the lowest target core temperature that a short-term temporising strategy should be implemented at is: *

- [ ] 32.0°C
- [ ] 33.0°C
- [ ] 34.0°C
- [ ] 35.0°C
- [ ] Other (please specify):

Comments:

20. In patients with exhausted intracranial volume buffering reserve and labile ICP with occasional spikes >25 mmHg, the lowest target core temperature that a medium term ICP-control strategy should be implemented at is: *

- [ ] 32.0°C
- [ ] 33.0°C
- [ ] 34.0°C
- [ ] 35.0°C
- [ ] Other (please specify):

Comments:

21. In tier 3 treatment, before considering decompressive craniectomy, hypothermia (<36.0C) should be attempted *

- [ ] True
- [ ] False

Comments:

22. Before considering barbiturate burst suppression, hypothermia (<36.0C) should be attempted *

- [ ] True
- [ ] False

Comments:

5. Fever

23. Neurogenic fever (core temperature >37.5°C driven by neurological dysregulation in the absence of sepsis or clinically significant inflammatory process) is relatively common in traumatic brain injury cases, and it should be promptly detected and treated (i.e., with controlled normothermia targeting 36.0°C to 37.5°C), irrespective of ICP. *

- [ ] True
- [ ] False
- [ ] Other (please specify):

Comments:

24. Controlled normothermia should be considered when pyrexia is secondary to sepsis or inflammatory processes, and when the patient is perceived to be at risk of secondary brain injury, especially in the acute phase of TBI. *

- [ ] True
- [ ] False
- [ ] Other (please specify):

Comments:

25. When fever is detected following severe TBI in cases perceived to be at risk of secondary brain injury, target temperature management should be initiated with a target core temperature range of 36.0°C to 37.5°C (i.e. controlled normothermia) *

- [ ] True
- [ ] False
- [ ] Other (please specify):

Comments:

26. In patients with severe TBI who are sedated and ventilated, controlled normothermia, irrespective of ICP, should be initiated:
*****

- [ ] Preventively
- [ ] Reactively when core temperature exceeds 36.5°C
- [ ] Reactively when core temperature exceeds 37.0°C
- [ ] Reactively when core temperature exceeds 37.5°C
- [ ] Reactively when core temperature exceeds 38.0°C
- [ ] Other (please specify):

Comments:

27. When neurogenic fever is detected in TBI cases, controlled normothermia should be continued for as long as the brain remains at risk of secondary brain damage *

- [ ] True
- [ ] False
- [ ] Other (please specify):

Comments:

6. TTM induction

28. It is recommended that the rapid induction of hypothermia in traumatic brain injury cases should be achieved with automated feedback-controlled temperature management devices *

- [ ] True
- [ ] False
- [ ] Other (please specify):

Comments:

29. It is advisable that neurotrauma ICUs should stock readily available NaCl solutions of different concentrations stored at ice-cold temperature for the management of intracranial hypertension crises *

- [ ] True
- [ ] False
- [ ] Other (please specify):

Comments:

7. TTM maintenance

30. An automated feedback-controlled TTM device that enables precise temperature control is desirable for the initiation of TTM and maintenance at target temperature in patients with severe TBI *

- [ ] True

☐ False

Comments:

31. The maximum temperature variation that a patient should experience during normothermia is less than or equal to +/- 0.5°C per hour and ≤1°C per 24-h period *

☐ True

☐ False

☐ Other (please specify):

Comments:

32. When hypothermia is indicated, treatment should be continued for as long as the brain is no longer considered to be at risk of secondary brain injury
*

☐ True

☐ False

☐ Other (please specify):

Comments:

8. TTM rewarming

33. Obtaining an interval scan and/or an alternative assessment of intracranial compliance, in addition to the absolute number of ICP, is recommended before rewarming *

- [ ] True
- [ ] False
- [ ] Other (please specify):

Comments:

34. When rewarming a pateint from mild hypothermia rewarming should be controlled by an automated feedback-controlled TTM device and should not exceed *

- [ ] a rate of ≤0.5C per 24-h period
- [ ] a rate of ≤1.0C per 24-h period
- [ ] a rate of ≤1.5C per 24-h period
- [ ] a rate of ≤2.0C per 24-h period
- [ ] Other (please specify):

Comments:

35. Rebound hyperthermia should be prevented whenever possible or promptly treated in cases when the brain is perceived to be at risk of secondary brain injury *

- [ ] True

☐ False

Comments:

9. Shivering

36. It is important to assess, document and manage shivering in severe TBI patients *

☐ True

☐ False

Comments:

37. Whenever ICP is labile and shivering is detected, neuromuscular blockers should be considered after ensuring appropriate depth of sedation
*****

☐ True

☐ False

☐ Other (please specify):

Comments:

38. In self-ventilating patients in the subacute phase of severe TBI, an individualised risk-benefit assessment should be undertaken regarding the indications of controlled normothermia. *

- [ ] True
- [ ] False

Comments:

39. Permissive hyperthermia should be considered in cases where risk of secondary brain injury resulting from pyrexia is thought to be low, and when shivering cannot be controlled with first line treatments such as NSAIDs, opiates, magnesium or counter warming
*

- [ ] True
- [ ] False
- [ ] Other (please specify):

Comments:

10. Auditing

40. Time within target range, burden of fever and similar metrics can be considered as indicators of quality of temperature management *

- [ ] True
- [ ] False

☐ Other (please specify): ____________________

Comments:
